# Epidemiological Features and Environmental Factors of Severe Fever with Thrombocytopenia Syndrome Patients in a Highly Endemic Region: A 12-Year Surveillance Study

**DOI:** 10.3390/pathogens15030328

**Published:** 2026-03-18

**Authors:** Xin Yang, Cheng-Juan Liu, Hong-Han Ge, Chun-Hui Li, Li-Fen Hu, Xiao-Ai Zhang, Ming Yue, Pei-Jun Guo, Wei Liu

**Affiliations:** 1 State Key Laboratory of Pathogen and Biosecurity, Academy of Military Medical Science, Beijing 100071, China; 17310598867@163.com (X.Y.); babylovehopi@163.com (X.-A.Z.); 2 Yantai Center for Disease Control and Prevention, Yantai 264003, China; 18865675969@163.com (C.-J.L.); lichunhui2015@163.com (C.-H.L.); 3 School of Public Health, Shandong First Medical University and Shandong Academy of Medical Sciences, Jinan 250117, China; ghh1129@126.com; 4 The First Affiliated Hospital of Anhui Medical University, Hefei 230032, China; lifen.hu@163.com; 5 Department of Infectious Diseases, The First Affiliated Hospital of Nanjing Medical University, Nanjing 210029, China

**Keywords:** severe fever with thrombocytopenia syndrome, long-term surveillance, tick, risk factors

## Abstract

**Background:** Severe fever with thrombocytopenia syndrome (SFTS) has become an increasing public health threat in China, with Yantai City representing a major endemic focus. A fine-scale, long-term epidemiological analysis integrating human case data with vector surveillance is essential for understanding local transmission dynamics. **Methods:** We conducted a retrospective analysis using 12-year (2013–2024) county-level SFTS surveillance data from Yantai City. Temporal trends were analyzed by Joinpoint regression. Concurrent field surveillance of *Haemaphysalis longicornis* (2019–2024) was used to quantify local SFTSV infection rates in ticks. Associations between SFTS incidence and environmental/livestock factors were evaluated using Spearman’s correlation and multivariable negative binomial regression. **Results:** A total of 1964 SFTS cases were reported. The annual incidence rate increased from 0.65 to 5.12 per 100,000 population, with an average annual percentage change (AAPC) of 13.56% 2013–2024, showing the most substantial rise among the elderly. Marked spatial heterogeneity was observed, with county-level mean incidence ranging from 0.30 to 5.23 per 100,000. The SFTSV infection rate in ticks surged from 0.54% in 2019 to 3.24% in 2024, and showed a strong positive correlation with human incidence both seasonally (ρ = 0.998) and across counties (ρ = 0.79), a pattern likely driven by shared environmental factors. Multivariable analysis identified grassland coverage (adjusted IRR [aIRR] = 1.21), woodland coverage (aIRR = 2.31), goat density (aIRR = 1.49), and tick infection rate (aIRR = 1.65) as independent risk factors, while urban land was protective (aIRR = 0.83). The overall case fatality rate was 8.86%, showing a declining trend, but was significantly higher in males (10.90%) than in females (7.04%), particularly among the elderly. **Conclusions:** SFTS incidence in Yantai increased significantly over the past decade, characterized by a heightened burden on the elderly and strong spatiotemporal clustering. Risk is independently mediated by ecological interfaces, notably woodland/grassland habitats and goat rearing. These findings delineate high-risk areas and populations, offering crucial insights for developing targeted public health strategies.

## 1. Introduction

Severe fever with thrombocytopenia syndrome (SFTS), caused by the *Bandavirus dabieense* (DBV) of the *Phenuiviridae* family, is an emerging tick-borne disease that poses a serious threat to global public health [1]. The disease is clinically characterized by acute febrile illness, gastrointestinal symptoms, leukopenia, and thrombocytopenia, with severe cases progressing to multi-organ dysfunction. Reported case fatality rates vary geographically, ranging from approximately 10% to 30% across endemic regions [2,3,4]. First identified in rural China in 2009, SFTS has since been reported in South Korea, Japan, Vietnam, Pakistan, Myanmar, and Thailand [5,6,7,8,9,10], with increasing incidence across East Asia over the past decade. In recognition of its growing public health threat, the World Health Organization (WHO) included SFTS among the top 10 priority infectious diseases requiring urgent research in 2017 [11].

The principal vector of DBV is the hard tick *Haemaphysalis longicornis*, which is native to East Asia and has established invasive populations in Oceania (e.g., Australia, New Zealand, and several Pacific Islands) and North America [12]. Climate change, particularly rising temperatures, is expanding suitable habitats for this vector, potentially accelerating its life cycle and widening its geographical distribution [13].

In China, SFTS has been a nationally notifiable disease since its discovery, with surveillance data collected through the China Information System for Disease Control and Prevention (CISDCP). Incidence has increased persistently over the last decade, with the virus now detected in more than 28 provinces. The distribution of *H. longicornis* across China closely mirrors that of human SFTS cases [14], underscoring the vector’s central role in transmission ecology. Accumulating evidence has demonstrated that environmental and meteorological factors profoundly influence SFTS transmission dynamics. Beyond climate factors, SFTS transmission is shaped by a complex interplay of ecological variables, including tick density, land cover, and habitat fragmentation, which collectively modify tick population dynamics and human–vector contact rates, influencing disease risk [15,16,17].

Despite these research findings, a critical knowledge gap remains. Previous studies have examined ecological associations at national or regional scales [16,17]. While useful for describing broad trends, such coarse spatial averaging may obscure the localized dose–response relationship between field-measured vector infection rates and human incidence. For a highly focal disease like SFTS, where transmission risk is modulated by micro-environmental variables such as microclimate, vegetation cover, and landscape connectivity that vary sharply at the county or village level [18,19,20], analyses at broader scales risk presenting an ecological fallacy. Specifically, the direct quantitative link between the SFTS virus (SFTSV) infection prevalence in *Haemaphysalis longicornis* ticks and the incidence of human cases at the local operational level, a cornerstone of entomological risk assessment, has not been systematically examined in high-endemic regions. This lack of fine-scale, integrated human–vector data has limited our understanding of local transmission drivers and hindered the development of precisely targeted interventions.

To address this gap, the present study analyzes 12 years of county-level surveillance data from Yantai City, one of China’s most highly endemic regions for SFTS [21,22,23]. We first characterize the demographic and temporal trends of SFTS incidence and fatality to identify vulnerable populations, and then test the hypothesis that ecological factors (e.g., land cover, livestock density) drive human risk not only directly, but also indirectly by shaping local viral circulation in the vector. To this end, we integrate field-measured SFTSV infection rates in H. longicornis ticks (2019–2024) to quantify the dose–response relationship between vector infection prevalence and human incidence at the county level, and identify the independent ecological determinants of local case clustering. By focusing on fine-scale spatial resolution and integrating concurrent human and vector surveillance data, this study aims to uncover localized transmission drivers that would be obscured in broader-scale analyses and to provide an evidence base for targeted interventions in high-risk areas.

## 2. Methods

### 2.1. Study Design and Data Collection

The study was performed in Yantai, Shandong province, a high-endemic region for SFTS. Located on the eastern coast of the Shandong Peninsula along the Yellow Sea, Yantai is characterized by a temperate monsoon climate, hilly terrain, and mixed agro-forestry ecosystems, which favor the proliferation of ticks.

Surveillance data for SFTS cases from 2013 to 2024 were obtained from the Yantai Center for Disease Control and Prevention (CDC). Collected variables included anonymized demographic information (age, sex, occupation), dates of illness onset and diagnosis, diagnostic method, clinical outcome and county-level residence. Case confirmation followed national SFTS guidelines [20] and required at least one of the following: (1) viral isolation or detection of SFTSV-RNA by reverse transcription-polymerase chain reaction (RT-PCR); (2) a ≥4-fold rise in SFTSV-specific antibody titers between acute and convalescent sera, measured by immunofluorescence assay (IFA), enzyme-linked immunosorbent assay (ELISA), or a neutralization test; (3) detection of SFTSV-specific IgM by IFA or ELISA.

County-level population data and domestic animal data (2013–2024) were obtained from the Yantai Municipal Statistical Yearbooks. Geospatial data, including administrative boundary vectors in polygon format and 1 km × 1 km land-use raster data, were obtained from the Resource and Environment Science and Data Center of the Chinese Academy of Sciences.

### 2.2. Tick Surveillance and Molecular Detection

Active tick surveillance was conducted at 52 sites across ten counties/districts of Yantai (Fushan, Haiyang, Laishan, Laiyang, Laizhou, Longkou, Muping, Penglai, Qixia and Zhaoyuan) from July 2019 to September 2024. Monthly collections were carried out from March to October, the active period for *H. longicornis*, yielding a total of 93 sampling dates: March (3), April (13), May (11), June (13), July (24), August (14), September (10), and October (5). No collections were made in January–February or November–December, due to tick inactivity. Collected ticks were morphologically identified under a stereomicroscope, separated by sex, and stored at −80 °C until further processing. For molecular detection, ticks were pooled by collection site and collection date, with a pool size ranging from 1 to 10 individuals (median: 5). Viral RNA was extracted from pooled ticks using a QIAamp Viral RNA Mini Kit (Qiagen, Hilden, Germany) and screened with a one-step, multiplex real-time RT-PCR assay (One Step PrimerScript RT-PCR Kit, Takara Bio, Shiga, Japan) targeting the SFTSV large (L) and small (S) segments. Positive samples were confirmed by conventional PCR with distinct primer sets, followed by Sanger sequencing and assembly using DNASTAR Lasergene (v7.0). Sequence identity was verified via BLAST against GenBank.

### 2.3. Statistical Analysis

Age-specific incidence rates were calculated using 5-year age intervals (e.g., 40–44, 55–59, 65–69 years), using the corresponding age-stratified mid-year population as the denominator. Due to limited sample size in finer age strata, individuals aged ≤39 years and those aged ≥75 years were each aggregated into a single group. For trend analysis, age was categorized into three groups (≤39, 40–59, and ≥60 years), and the study period (2013–2024) was divided into four 3-year intervals (2013–2015, 2016–2018, 2019–2021, and 2022–2024). Age- and sex-specific incidence trends were analyzed using the Joinpoint Regression Program (v4.9.1.0, NCI) to identify inflection points and estimate annual percentage changes (APCs) and average APCs (AAPC) based on Poisson distributed generalized linear models. County-level mean incidence (per 100,000 population on mid-year) and APCs were calculated to compare regional trends.

To align with the tick surveillance period, county-level human SFTS incidence for 2019–2024 was calculated as the mean annual incidence per 100,000 population. All analyses incorporating tick infection data were restricted to the overlapping period to ensure temporal consistency between human and vector surveillance. The SFTSV infection rate in *H. longicornis* ticks was estimated using maximum likelihood estimation (MLE) implemented in PooledInfRate (v4.0), which accounts for pooled testing and provides prevalence estimates with 95% confidence intervals. For seasonal correlation analysis, mean tick infection rates and mean human incidence were calculated for each season (spring, summer, autumn) by averaging data from 2019 to 2024, yielding three paired observations. The correlation between tick infection rate and human incidence was assessed using Spearman’s rank correlation (ρ).

To evaluate potential spatial dependence of SFTS incidence, global spatial autocorrelation was assessed at the county level using Moran’s *I*. Spatial weights were defined based on queen contiguity (counties sharing boundaries or vertices) and statistical significance was evaluated using 999 Monte Carlo permutations.

Thirteen county-level variables were examined as potential environmental determinants: seven land-cover types (dry land, woodland, grassland, water body, urban land, rural residential areas, orchard), elevation, distance to bird habitats, population density, goat density, cattle density and the SFTSV infection rate in *H. longicornis* ticks. Univariate associations with average annual SFTS incidence were evaluated using Spearman’s correlation. Crude incidence rate ratios (IRRs) were obtained from univariate negative binomial regression; variables with *p* < 0.05 were retained for inclusion in a multivariable negative binomial model. Model diagnostics included assessment of overdispersion via a likelihood ratio test comparing the negative binomial model to a Poisson model. Multicollinearity among explanatory variables was evaluated using variance inflation factors (VIFs). For descriptive purposes, continuous variables were categorized into tertiles to illustrate average SFTS incidences across different exposure levels in tabular presentations (this categorization was employed solely to enhance the interpretability of descriptive statistics and to visualize trends across low, medium, and high exposure categories); all regression analyses retained continuous variables in their original form to preserve statistical power and avoid obscuring potential dose–response relationships.

Continuous variables were presented as median (interquartile range, IQR), and compared using the Mann–Whitney U test (for two groups) or the Kruskal–Wallis test (≥3 groups). Categorical variables were summarized as counts (%) and compared using the chi-square (χ^2^) test. All statistical analyses were performed using R version 4.1.2 (R Foundation for Statistical Computing, Vienna, Austria). A two-sided *p* value < 0.05 was considered statistically significant.

## 3. Results

### 3.1. Overall Epidemiological Characteristics of SFTS Cases

Between 2013 and 2024, a total of 1964 cases of SFTS were reported in Yantai City, with 99.13% (1947/1964) being laboratory-confirmed (Table 1). The overall incidence over this period was 2.26 per 100,000 population. Among the reported cases, 52.80% (1037/1964) were female and 80.50% (1581/1964) were farmers (Table 1). The median age of patients was 67 years (IQR: 58–74), increasing from 62 years in 2014 to 68 years in 2024 (Supplementary Figure S1A). Seasonal variation in median age was also noted, with the highest value in September (68 years) and the lowest in March (52 years) (Supplementary Figure S1B).

**Figure 1:**
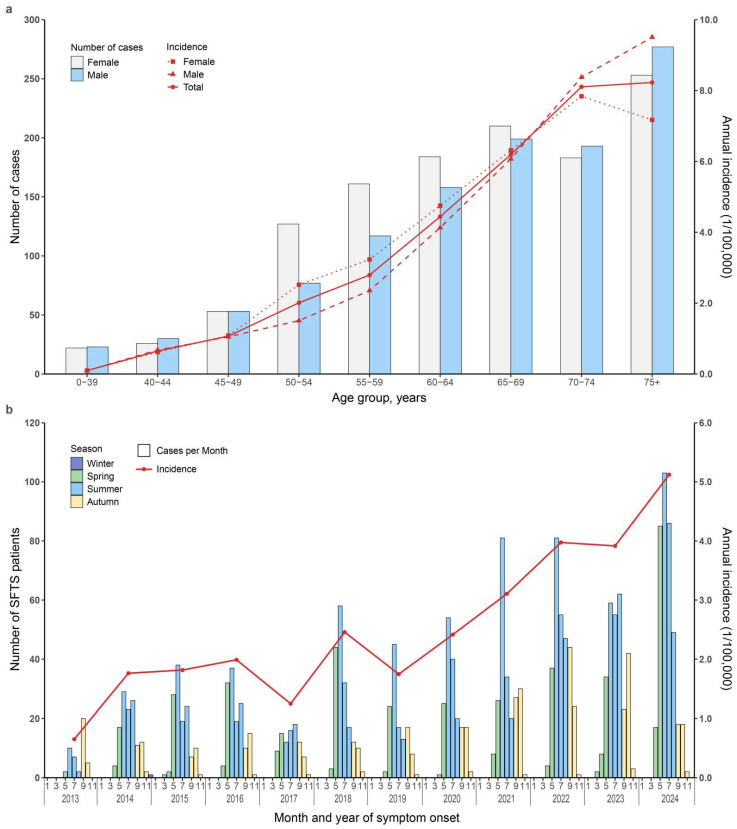
Epidemiological characteristics of SFTS cases in Yantai City, 2013–2024. (**a**) Number of cases and annual incidence rate. The panel combines a bar chart (left axis) for case counts by age group and sex with a line chart (right axis) for the corresponding annual incidence rates per 100,000 population. The ‘75+’ category represents individuals aged 75 years or older. (**b**) Temporal distribution of SFTS cases. The panel shows the temporal distribution of SFTS cases. Seasons were defined as: Winter (December, January, February); Spring (March, April, May); Summer (June, July, August); Autumn (September, October, November).

Incidence rate was higher in females than in males, particularly among individuals aged 50–70 years (Figure 1a). From 2013 to 2024, the annual incidence increased nearly eightfold, from 0.65 per 100,000 population in 2013 to 2.46 in 2018, and further to 5.12 in 2024, corresponding to a significant average annual increase of 13.56% (95% CI: 9.63–19.35%) over the study period (Figure 1b). A distinct seasonal pattern was evident, with most cases occurring in summer, followed by autumn and spring, while incidence approached zero during winter (Figure 1b). The median time from symptom onset to hospital admission was 7 days (IQR: 5–11), which decreased over time, from 12 days (IQR: 7–19) in 2013 to 6 days (IQR: 4–7) in 2024 (Supplementary Figure S2A). Seasonal variation was also noted, with the longest delays in April (9 days, IQR: 7–13) and the shortest in October (7 days, IQR: 4–11) (Supplementary Figure S2B). The overall case fatality rate was 8.86% (174/1964), declining from 13.04% in 2013 to 5.60% in 2024 (Table 1 and Supplementary Table S1).

### 3.2. Long-Term Trends in Case Incidence by Age and Sex

**Figure 2:**
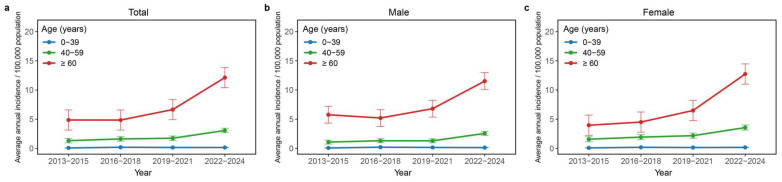
Trends in age-specific average annual incidence of SFTS in Yantai City across four time periods. The figure consists of three panels showing incidence rates for total patients (**a**), male patients (**b**) and female patients (**c**). For each panel, the average annual incidence (per 100,000 population) is plotted for three age groups (0–39, 40–59, and ≥60 years) across four consecutive 3-year periods (2013–2015, 2016–2018, 2019–2021, and 2022–2024). Data points represent mean incidence rates and bars indicate 95% confidence intervals.

Analysis of age- and sex-specific incidence trends across four consecutive periods (2013–2015, 2016–2018, 2019–2021, and 2022–2024) revealed a consistent age-dependent increase, with the most pronounced elevation observed among individuals aged 40–59 years and ≥60-year years (Figure 2a–c). Incidence rates in the most recent period (2022–2024) were significantly higher than in all preceding periods across every age group and both sexes, indicating a sustained and broad increase over time. This upward trend was most pronounced among older individuals. For example, incidence in the ≥60 age group more than doubled from 4.87 per 100,000 in 2013–2015 to 12.13 per 100,000 in 2022–2024. Age-stratified analysis identified the highest annual percent change (APC) in patients aged ≥60 years (APC 16.17%, 95% CI: 9.97–26.64), followed by those aged 40–59 years (APC 12.59%, 95% CI: 6.18–22.13). In contrast, no significant trend was observed in the 0–39 years age group (APC of 2.91; 95% CI: −12.13–20.31) (Supplementary Table S2).

Sex-specific analysis further revealed distinct temporal patterns. Among males aged 40–59 years, incidence exhibited considerable fluctuation, with a significant increase during 2013–2015 (APC 16.60%, 95% CI: 5.44–44.39), a non-significant decline from 2015 to 2018, and a sharp rise from 2018 onward (APC 21.91%, 95% CI: 14.84–50.72). Males aged ≥60 years also demonstrated a significant upward trend (APC 13.39%, 95% CI: 4.98–26.85). Among females, those aged 0–39 years exhibited a sharp increase from 2013 to 2017 (APC: 50.66%, 95% CI: 12.37–92.71), followed by a significant decline from 2017 to 2024 (APC: −12.70%, 95% CI: −27.34 to −4.21). In contrast, females aged 40–59 and ≥60 years showed steadily rising trends, with the highest APC observed in the ≥60 age group (19.17%, 95% CI: 12.92–30.13) (Supplementary Table S2).

### 3.3. Seasonal Trends in Case Incidence by Age and Sex

A consistent seasonal peak was observed between May and July, accounting for an average of 60.57% of annual cases. Although this proportion varied across years, ranging from 41.30% in 2013 to 75.28% in 2018, it remained between 60% and 70% in most years (Supplementary Figure S3).

Significant increasing trends (AAPC > 0) were observed during March–April (17.15%; 95% CI: 6.23–37.51), May–June (15.87%; 95% CI: 5.51–32.96), July–August (16.56%; 95% CI: 10.32–27.30), and September–October (15.63%; 95% CI: 7.85–23.98). In contrast, no significant trend was detected for November–December (AAPC: 1.48%; 95% CI: −22.62–33.07) (Supplementary Figure S4).

Monthly incidence exhibited a strong age-dependent pattern (Supplementary Figure S5A). Young adults (0–39 years) showed consistently low incidence rates (0–0.04 per 100,000), with slight peaks in May and July. Both middle-aged (40–59 years) and elderly (≥60 years) groups displayed similar seasonal trends: incidence increased from March to June, peaked in June (0.41 and 1.75 per 100,000, respectively), and subsequently declined through November.

Seasonal patterns also varied by sex (Supplementary Figure S5B). Throughout the study period, females exhibited consistently higher monthly incidence rates than males in most months, with the exception of in July and September, when rates were comparable. The sex-based disparity was most pronounced during the peak transmission period from May to July. Notably, incidence among females in May (0.54 per 100,000) was nearly twice that of males (0.29 per 100,000), and remained elevated in June (0.75 vs. 0.62 per 100,000). Both sexes followed similar seasonal trends, with incidence rising from April, reaching a peak in June, and declining thereafter. The absolute increase in cases during the seasonal peak was greater in females, aligning with the overall higher disease burden observed in this group.

### 3.4. Spatiotemporal Patterns of SFTS Incidence

**Figure 3:**
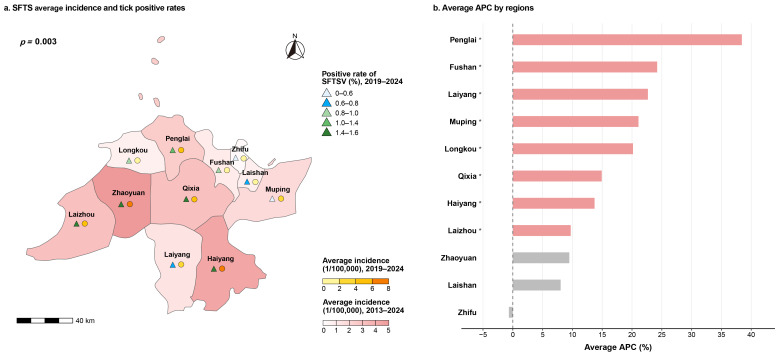
Regional distribution of the SFTS average annual incidence rates and results of the Joinpoint trend analysis by regions. (**a**) SFTS average annual incidence rates and tick positive rates. The pink gradient represents the average annual SFTS incidence in Yantai City from 2013 to 2024, and the yellow gradient represents the average annual incidence from 2019 to 2024. The triangles indicate the positive rate of SFTSV in *Haemaphysalis longicornis* ticks. County names are labeled on the map. (**b**) Average APC by regions in Yantai City, 2013–2024. APC, annual percentage change. Asterisks (*) and Pink bars represent statistically significant APC estimates (*p* < 0.05), while grey bars represent non-significant trends.

Between 2013 and 2024, SFTS cases in Yantai geographically expanded from an initial six counties to all 11 counties (districts), reflecting progressive geographic distribution. County-level mean incidence rates varied considerably, with the highest rates observed in Zhaoyuan (5.23 per 100,000), Haiyang (4.60 per 100,000), and Qixia (3.22 per 100,000), and the lowest in Zhifu (0.30 per 100,000), Laishan (0.87 per 100,000), and Fushan (1.04 per 100,000) (Figure 3a). Spatial autocorrelation analysis using Moran’s *I* revealed significant positive spatial dependence of SFTS incidence across counties (Moran’s *I* = 0.32, *p* < 0.05). Joinpoint regression analysis revealed significant increasing trends in eight counties. Penglai exhibited the highest average annual percent change (AAPC) at 38.43% (95% CI: 32.89–49.91), followed by Laiyang (22.67%, 95% CI: 16.24–29.45), Fushan (24.22%, 95% CI: 16.21–39.93), Muping (21.09%, 95% CI: 12.68–35.95), Longkou (20.17%, 95% CI: 8.63–41.10), Qixia (14.92%, 95% CI: 5.05–32.24), Haiyang (13.72%, 95% CI: 5.06–27.39), and Laizhou (9.72%, 95% CI: 5.69–13.89). Notably, Laizhou displayed a biphasic pattern, characterized by a rapid initial increase from 2013 to 2016 (APC: 35.05%), and followed by a slower rise thereafter (APC: 6.29%). Conversely, Laiyang exhibited a significant acceleration after 2018 (APC: 31.92%) following a period of stable incidence. The remaining three counties showed no statistically significant trends during the study period (Table 2, Figure 3b).

### 3.5. Association Between Environmental Factors, Tick Infection, and Human SFTS Incidence

To identify county-level ecological correlates of SFTS incidence, we employed Spearman correlation and negative binomial regression analyses. Univariate correlation analysis indicated that incidence was positively correlated with elevation (ρ = 0.845), goat density (ρ = 0.773), grassland coverage (ρ = 0.718), and SFTSV infection rates in *H. longicornis* ticks (ρ = 0.790), while negatively correlated with population density (ρ = −0.791) and urban land cover (ρ = −0.755) (all *p* < 0.05). No significant correlations were observed for orchard coverage, cattle density, woodland coverage, rural residential area coverage, distance to migratory bird habitats, dryland coverage, or water body coverage (Supplementary Figure S6). In univariate negative binomial regression analysis, eight variables were significantly associated with incidence: coverage of woodland, grassland, urban land, rural residential areas, elevation, population density, goat density, and SFTSV infection rates in *H. longicornis* ticks (all *p* < 0.05). These variables were subsequently included in a multivariate model, which identified five independent predictors (Supplementary Table S3). The likelihood ratio test confirmed significant overdispersion (*p* < 0.001), supporting the use of negative binomial regression over Poisson regression. Variance inflation factors for all explanatory variables were below 5 (range: 1.26–4.89), indicating no substantial multicollinearity.

In the multivariable analysis, the following county-level factors were independently associated with SFTS incidence: a 10% increase in grassland coverage was associated with a 21% increase in incidence (adjusted IRR [aIRR] = 1.21, 95% CI: 1.13–1.47; *p* = 0.006). Woodland coverage exhibited an even stronger positive association (aIRR = 2.31, 95% CI: 1.28–4.16; *p* = 0.028). For each increment of 10 goats per km^2^, incidence increased by 49% (aIRR = 1.49, 95% CI: 1.12–1.76; *p* = 0.016). Notably, a 1% rise in the tick SFTSV infection rate was associated with a 65% increase in human incidence (aIRR = 1.65, 95% CI: 1.12–2.43; *p* = 0.011). In contrast, a 10% increase in urban land cover corresponded to a 17% reduction in incidence (aIRR = 0.83, 95% CI: 0.67–0.96; *p* = 0.036) (Supplementary Table S3).

### 3.6. Temporal and Spatial Association Between Tick Infection and Human Incidence

The strong association identified by the regression model was further examined across temporal and spatial scales. Analysis of 12,155 *H. longicornis* ticks collected between 2019 and 2024 revealed synchronized seasonal patterns between tick SFTSV infection rates and human incidence. Both measures peaked in summer (1.71% and 2.091 per 100,000, respectively; Spearman’s ρ = 0.998, *p* = 0.037), followed by spring (0.54% and 0.620 per 100,000) and autumn (0.51% and 0.680 per 100,000), with a strong positive correlation (Spearman’s ρ = 0.998, *p* = 0.037; Supplementary Figure S7A). At the annual scale, both measures increased concurrently from 2019 to 2024. Human incidence rose from 1.75 to 5.12 per 100,000, while tick infection rate increased from 0.54% to 3.24% (AAPC = 52.20%, 95% CI: 21.25–75.34), demonstrating a strong temporal correlation (Spearman’s ρ = 0.957, *p* = 0.003; Supplementary Figure S7B).

Spatially, tick infection rates varied considerably among counties, ranging from 0% in Zhifu to 1.58% in Zhaoyuan, with elevated rates also observed in Laizhou (1.47%), Qixia (1.49%), and Haiyang (1.56%). A strong positive correlation was observed between county-level tick infection rates and human incidence (Spearman’s ρ = 0.790, *p* = 0.003; Figure 3a).

### 3.7. Temporal Trends of Case Fatality by Age and Sex

The overall case fatality rate (CFR) was 8.86% (174/1964). During the study period, CFR declined from 13.04% in 2013 to 5.60% in 2024 (Supplementary Table S1). No significant seasonal variation was detected, with comparable CFRs observed during the high-incidence season (May–July) and the remainder of the year (January–April, August–December). The median age of fatal cases was 70 years (IQR 63–75), exhibiting a clear upward trend over time, increasing from 59 years in 2013 to 75 years by 2024 (Supplementary Figure S8). Males accounted for approximately 58% of fatal cases overall, with annual proportions ranging from 33.3% to 70.0%. In earlier years (e.g., 2013), females constituted a notably higher proportion of fatal cases (66.7%); however, this proportion gradually decreased over time. By 2024, the sex distribution approached parity (45% female vs. 55% male), indicating a potential shift in epidemiological patterns or risk factors (Supplementary Figure S8).

CFR increased with age, from 0% in the 0–39 years group to 5.05% in the 40–59 years group, peaking 10.44% in the ≥60 years group. This age-dependent pattern remained consistent throughout the study period (Supplementary Figure S9). Overall, CFR was significantly higher among males than females (10.90% vs. 7.04%, *p* = 0.003). The disparity was most marked in the elderly (≥60 years), with a CFR of 12.92% for males compared to 8.50% for females (*p* = 0.009). No statistically significant difference was observed in younger age groups (40–59 years) (5.86% for males vs. 4.09% for females) (Supplementary Table S4).

## 4. Discussion

This study documents a persistent and growing burden of SFTS in Yantai, a major endemic focus in China, over 12 years (2013–2024). By integrating long-term human surveillance data, concurrent field investigation of tick infection, and fine-scale ecological niche modelling, we elucidate the evolving epidemiology and localized ecological drivers of SFTS risk. The county-level analytical framework preserved local heterogeneity and enabled detection of risk gradients that would likely be obscured in broader-scale analyses, underscoring the value of fine spatial resolution for understanding SFTS transmission dynamics.

Consistent with national surveillance trends, we observed a marked and sustained increase in human SFTS incidence, with an average APC of 13.56%. This upward trajectory likely reflects not only enhanced surveillance but also a concurrent sharp increase in local tick infection rates, which increased from 0.54% in 2019 to 3.24% in 2024. The strong spatiotemporal correlation between these measures may suggest an intensification of the enzootic transmission cycle. Underlying mechanisms may include climate warming, which prolongs tick activity periods and enhances reproductive rates, coupled with potential increases in reservoir host densities [24]. Together, these factors could expand the environmental pathogen reservoir and elevate spillover risk to humans [24].

Despite increasing incidence, the overall CFR showed a decreasing trend, suggesting improvements in clinical awareness, diagnostic capabilities, and supportive care. Nevertheless, the demographic characteristics of fatal cases shifted, with increases in both median age and the proportion of males. The disease burden predominantly affected older adults (≥60 years) and farmers (accounting for over 80% of cases), a pattern recognized as a hallmark of SFTS [25]. Notably, temporal analysis disclosed the most rapid increase in incidence among individuals aged ≥75 years. However, while incidence declined among elderly females aged ≥75 years, it remained persistently high among elderly males. The sex distribution of fatalities shifted from a female predominance in the early years to near-equality in recent years. These patterns might reflect a combination of behavioral and biological factors. As women age, they often transition from high-risk outdoor activities toward domestic roles, potentially reducing exposure to tick habitats, whereas older males frequently continue outdoor labor, maintaining higher exposure levels [25]. Age-related immune decline (known as immunosenescence) may also contribute, with evidence suggesting that aging males experience more rapid immune deterioration (reduced T-cell response, increased inflammation), a phenomenon also observed in COVID-19 [26,27,28].

As previously demonstrated in SFTS, immune decline associated with aging was more pronounced in males than in females [29]. Although our study lacked direct measurements of immune function, these biological factors may explain the steeper age-specific gradient in CFR among males and the disproportionately higher mortality in older men. Incidence varied substantially across counties, with high-risk regions such as Zhaoyuan and Haiyang contrasting sharply with low-risk urban areas like Zhifu. Multivariable analysis identified several key ecological determinants: (1) Habitat suitability: High woodland and grassland coverage provides a favorable microclimate (shade, moisture) that is essential for the survival and questing activity of moisture-sensitive *H. longicornis*, and supports abundant wildlife hosts, which sustain dense tick populations [30,31]. (2) Agricultural amplification: Goat density emerged as a stronger independent risk factor than cattle density. Goats, being highly competent feeding hosts for *H. longicornis,* promote prolific tick reproduction [32]. Free-ranging goats likely serve as bridging amplifiers, as they forage at woodland-grassland interfaces where they acquire infected ticks, then transport them back to peridomestic areas, simultaneously boosting tick populations through feeding and amplifying viral circulation via co-feeding or systemic infection, thus enhancing tick abundance and virus circulation in peri-domestic areas and thereby increasing human exposure [33]. In contrast, urban land cover was protective due to its unsuitability for tick survival [34]. The significant spatial autocorrelation of SFTS incidence (Moran’s *I* = 0.32, *p* < 0.05) aligns with the clustered spatial distribution of these environmental risk factors, which suggests that the observed spatial clustering is not random, but likely driven by the underlying heterogeneity in ecological landscapes—particularly the concentration of woodland/grassland coverage and goat density in specific counties. These factors create localized environmental ‘hotspots’ that perpetuate the tick-host transmission cycle. This finding underscores the need for geographically targeted interventions in high-risk clusters such as Zhaoyuan and Haiyang.

The near-perfect seasonal correlation (ρ = 0.998) between tick infection rates and human incidence warrants cautious interpretation. In temperate regions, *H. longicornis* activity is largely confined to the period from March to October, with minimal activity during winter months due to low temperatures [35]. This phenological pattern drives the seasonal availability of host-seeking ticks and, consequently, the temporal variation in human exposure risk [35]. Similarly, tick infection rates fluctuate with active host-seeking behavior, as viral transmission is contingent upon tick feeding [31]. Therefore, the observed correlation likely reflects a shared dependence on seasonal drivers. Moreover, the limited number of seasonal data points may have inflated the correlation coefficient.

A key finding is the dose–response relationship between the SFTSV infection rate in questing ticks and human incidence at the county level. This entomological risk index establishes a critical mechanistic connection between environmental suitability (such as landscape and host availability) and human disease outcomes. Higher densities of infected ticks elevate the per-bite transmission risk [36], providing a biological explanation for spatial variations in risk, though whether this pattern persisted in earlier years remains unknown.

Several limitations should be acknowledged. First, surveillance data may not fully capture mild cases, potentially introducing bias into risk estimates. Second, although county-level ecological analysis provides useful insights, it might mask risk factors operating at finer scales, such as those at the village or household level. Third, tick surveillance was confined to the period from 2019 to 2024, restricting tick-related analyses to this timeframe and preventing evaluation of earlier associations between tick presence and disease incidence. This period also coincided with the COVID-19 pandemic (2020–2023), when public health measures may have affected exposure risk or case identification. Fourth, the presence of significant spatial autocorrelation may lead to potential bias in standard errors due to spatial dependence. Future studies with larger sample sizes should consider using spatial regression models to address this. Additionally, the limited number of spatial units relative to candidate predictors introduces overfitting risk. Although we reduced predictors to five after screening and confirmed no substantial multicollinearity, the suboptimal observation-to-predictor ratio warrants cautious interpretation. Furthermore, unmeasured confounders, including rodent density and specific agricultural practices, could influence the results. However, the SFTSV infection rate derived from intensive testing of *H. longicornis* may help mitigate such biases.

Our findings reveal a rapidly escalating public health threat posed by SFTS, characterized by distinct demographic vulnerabilities, pronounced spatiotemporal clustering, and specific environmental drivers. In light of this evidence, targeted tick control in high-risk environments, such as woodlands, grasslands, and goat farming areas, could prove beneficial. Recommended measures include acaricide application and clearing vegetation near residential zones. It is also advisable to prioritize protection for elderly farmers during the high-risk period (April–October) through personal protective measures and community health education, which could help reduce disease burden. Furthermore, incorporating tick infection surveillance into early-warning systems could enable proactive responses. Future research should focus on integrated vector–livestock management approaches, assess the cost-effectiveness of habitat modification, and evaluate behavioral interventions via community trials. Longitudinal serological studies and viral sequencing of human-tick-animal samples are also warranted to elucidate transmission dynamics and viral evolution.

## 5. Conclusions

This 12-year study conducted in Yantai, a major endemic focus of SFTS, documents a significant and sustained increase in human incidence (AAPC = 13.56%) alongside a declining case fatality rate. The shift toward older and more balanced sex distribution among fatal cases suggests growing vulnerability in elderly males, potentially associated with sex-related immunosenescence. Critically, integrating human surveillance with parallel entomological monitoring revealed a clear dose–response relationship between the SFTSV infection rate in *H. longicornis* and county-level human incidence during 2019–2024. The sharp increase in tick infection prevalence during this period coincided with rising human case numbers, a pattern indicative of an intensified enzootic cycle. Spatial heterogeneity in SFTS risk was independently influenced by woodland and grassland coverage, goat density, and local tick infection rates, while urban land appeared protective. These findings underscore the critical role of ecological interfaces in SFTS transmission. From a public health perspective, this evidence supports targeted tick management in high-risk agro-pastoral landscapes, prioritized protection for elderly farmers during seasonal outbreaks, and the incorporation of tick infection data into surveillance systems for early warning. In summary, our study advances the understanding of SFTS transmission dynamics at a fine spatial scale and offers an evidence-based foundation for designing targeted interventions in highly endemic regions.

## Figures and Tables

**Table 1:** Demographic characteristics and outcome of laboratory-confirmed and clinically confirmed SFTS cases.

Characteristics	Total (N = 1964)	Laboratory-Confirmed Cases (N = 1947)	Clinically Confirmed Cases (N = 17)	*p*
Age [years, median (IQR)]	67 (58, 74)	67 (59, 74)	56 (53, 68)	0.021
Gender (n, %)				1.000
Male	927 (47.20%)	919 (47.20%)	8 (47.06%)	
Female	1037 (52.80%)	1028 (52.80%)	9 (52.94%)	
Occupation (n, %)				0.179
Farmer	1581 (80.50%)	1570 (80.64%)	11 (64.71%)	
Non-farmer	383 (19.50%)	377 (19.36%)	6 (35.29%)	
Delay from onset to hospital [days, median (IQR)]	7 (5, 11)	7 (5, 11)	6 (5, 8)	0.207
Outcome (n, %)				0.394
Fatal	174 (8.86%)	171 (8.78%)	3 (17.65%)	
Survival	1790 (91.14%)	1776 (91.22%)	14 (82.35%)	

Note: Data are n (%) unless otherwise specified. Categorical variables were compared between groups using χ^2^ tests. Continuous variables were presented as medians with interquartile ranges (IQRs) and were compared using the Mann–Whitney U test. *p* values less than 0.05 were considered statistically significant.

**Table 2:** The trend changes in SFTS incidence by regions in Yantai, 2013–2024.

Regions	Total Cases of SFTS	Period 1		Period 2		Entire Study Period
		Years	APC (95% CI)	Years	APC (95% CI)	Average APC (95% CI)
Haiyang	365	2013–2024	13.72 # (5.06–27.39)			13.72 # (5.06–27.39)
Qixia	236	2013–2024	14.92 # (5.05–32.24)			14.92 # (5.05–32.24)
Zhaoyuan	367	2013–2024	9.48 (−1.09–25.29)			9.48 (−1.09–25.29)
Laizhou	355	2013–2016	35.05 # (15.47–91.02)	2016–2024	6.29 (−1.31–9.66)	9.72 # (5.69–13.89)
Laiyang	157	2013–2018	5.45 (−38.05–60.92)	2018–2024	31.92 # (9.71–72.67)	22.67 # (16.24–29.45)
Longkou	67	2013–2024	20.17 # (8.63–41.10)			20.17 # (8.63–41.10)
Penglai	159	2013–2024	38.43 # (32.89–49.91)			38.43 # (32.89–49.91)
Laishan	38	2013–2018	73.77 # (38.48–187.93)	2018–2024	−9.06 (−32.01–0.73)	8.03 (−7.67–26.43)
Muping	111	2013–2024	21.09 # (12.68–35.95)			21.09 # (12.68–35.95)
Fushan	79	2013–2024	24.22 # (16.21–39.93)			24.22 # (16.21–39.93)
Zhifu	30	2013–2024	−0.66 (−16.11–19.17)			−0.66 (−16.11–19.17)

Note: # Regions with a significantly increasing trend. SFTS, severe fever with thrombocytopenia syndrome; CI, confidence interval; APC, annual percentage change.

## Data Availability

The original contributions presented in the study are included in the article, and further inquiries can be directed to the corresponding authors.

## Supplementary Materials

The following supporting information can be downloaded at: https://www.mdpi.com/article/10.3390/pathogens15030328/s1, Supplementary Table S1. Demographic and epidemiological characteristics between survived and deceased SFTS patients. Supplementary Table S2. Joinpoint trend analysis of SFTS in Yantai, by age group and sex, 2013–2024. Supplementary Table S3. The risk factors associated with SFTS incidence identified by the negative binomial regression model at the county level. Supplementary Table S4. Age- and sex-stratified case numbers and fatality rates of SFTS patients in Yantai, 2013–2024. Supplementary Figure S1. Median age of SFTS cases in Yantai City. Supplementary Figure S2. The delay days between disease onset and admission of SFTS cases in Yantai City. Supplementary Figure S3. Monthly distribution of SFTS cases in every year. Supplementary Figure S4. Average Annual Percent Change (APC) of SFTS incidence by bimonthly season in Yantai City, 2013–2024. Supplementary Figure S5. Monthly trends in SFTS incidence by age group and sex in Yantai City, 2013–2024. Supplementary Figure S6. Correlation analysis between average SFTS incidence and environmental factors in Yantai City. Supplementary Figure S7. Temporal association between human SFTS incidence and SFTSV infection rate in H.longicornis ticks in Yantai City, 2019–2024. Supplementary Figure S8. Annual demographic characteristics of fatal SFTS cases in Yantai City, 2013–2024. Supplementary Figure S9. Annual case-fatality rates of SFTS by age group in Yantai City, 2013–2024.
