# Supplementary material for: Epidemiological Features and Environmental Factors of Severe Fever with Thrombocytopenia Syndrome Patients in a Highly Endemic Region: A 12-Year Surveillance Study
Source: Pathogens. 2026 Mar 18;15(3):328. doi: 10.3390/pathogens15030328 (PMC13029095; doi:10.3390/pathogens15030328)
Supplement: Supplementary file 1 [file pathogens-15-00328-s001.zip › Supplemental materials.pdf]

**Supplementary Table S1. Demographic and epidemiological characteristics between survived and deceased SFTS patients.**

| Characteristics | Total (N=1964) | Survival cases (N=1790) | Death cases (N=174) | *P* |
|---|---|---|---|---|
| Age [years, median (IQR)] | 67 (58, 74) | 66 (58, 74) | 70 (63, 75) | <0.001 |
| Gender (n, %) | | | | 0.003 |
| Male | 927 (47.20%) | 826 (46.15%) | 101 (58.05%) | |
| Female | 1037 (52.80%) | 964 (53.85%) | 73 (41.95%) | |
| Occupation (n, %) | | | | 0.626 |
| Farmer | 1581 (80.50%) | 1438 (80.34%) | 143 (82.18%) | |
| Non-farmer | 383 (19.50%) | 352 (19.66%) | 31 (17.82%) | |
| Delay from onset to hospital [days, median (IQR)] | 7 (5, 11) | 7 (5, 11) | 9 (6, 12) | <0.001 |
| Temporal distribution (n, %) | | | | <0.001 |
| 2013 | 23 (1.17%) | 20 (1.12%) | 3 (13.04%) | |
| 2014 | 91 (4.63%) | 81 (4.53%) | 10 (10.99%) | |
| 2015 | 111 (5.65%) | 98 (5.47%) | 13 (11.71%) | |
| 2016 | 122 (6.21%) | 114 (6.37%) | 8 (6.56%) | |
| 2017 | 82 (4.18%) | 72 (4.02%) | 10 (12.20%) | |
| 2018 | 137 (6.98%) | 118 (6.59%) | 19 (13.87%) | |
| 2019 | 108 (5.50%) | 94 (5.25%) | 14 (12.96%) | |
| 2020 | 157 (7.99%) | 140 (7.82%) | 17 (10.83%) | |
| 2021 | 218 (11.10%) | 194 (10.84%) | 24 (11.01%) | |
| 2022 | 280 (14.26%) | 260 (14.53%) | 20 (7.14%) | |
| 2023 | 278 (14.15%) | 262 (14.64%) | 16 (5.76%) | |
| 2024 | 357 (18.18%) | 337 (18.83%) | 20 (5.60%) | |
| Seasonality [from May to July] | 1244 (63.34%) | 1125 (62.85%) | 119 (68.39%) | 0.172 |

Note: Data are n (%) unless otherwise specified. In the "Temporal distribution" section, the "Death cases" column displays both the number of deaths and the annual case fatality rate (calculated as [death cases / total cases] × 100% for that year). Categorical variables were compared between groups using $\chi 2$ tests. Continuous variables were presented as medians with interquartile ranges (IQR) and were compared using the Mann-Whitney U test. *P* values less than 0.05 were considered statistically significant.

**Supplementary Table S2. Joinpoint trend analysis of SFTS in Yantai, by age group and sex, 2013–2024.**

| Age group (years) | Year | APC (95% CI) |
|---|---|---|
| **All patients** | 2013–2024 | 13.56# (9.63–19.35) |
| 0-39 | 2013–2024 | 2.91 (-12.13–20.31) |
| 40-59 | 2013–2024 | 12.59# (6.18–22.13) |
| ≥60 | 2013–2024 | 16.17# (9.97–26.64) |
| **Male patients** | | |
| 0-39 | 2013–2024 | 4.58 (-2.83–12.82) |
| 40-59 | 2013–2015 | 16.60# (5.44–44.39) |
| 40-59 | 2015–2018 | -13.22 (-25.12–5.48) |
| 40-59 | 2018–2024 | 21.91# (14.84–50.72) |
| ≥60 | 2013–2024 | 13.39# (4.98–26.85) |
| **Female patients** | | |
| 0-39 | 2013–2017 | 50.66# (12.37–92.71) |
| 0-39 | 2017–2024 | -12.70# (-27.34– -4.21) |
| 40-59 | 2013–2024 | 11.68# (3.70–23.42) |
| ≥60 | 2013–2024 | 19.17# (12.92–30.13) |

Note: #Categories with a significantly increasing trend. SFTS, severe fever with thrombocytopenia syndrome; CI, confidence interval; APC, annual percentage change.

**Supplementary Table S3. The risk factors associated with SFTS incidence identified by the negative binomial regression model at the county level.**

| Variable (Type, unit) | Annual average incidence (95% CI, per 100,000 person-years) | Univariate analysis Crude IRR (95% CI) | *P* | Multivariate analysis Adjusted IRR (95% CI) | *P* |
|---|---|---|---|---|---|
| Dry land (categorical, 1%) | | | | | |
| <41.38 | 1.61 (0.96, 2.26) | | | | |
| 41.38-56.99 | 3.45 (2.65, 4.25) | | | | |
| >56.99 | 1.91 (1.41, 2.41) | | | | |
| Dry land (continuous, 10%) | | 1.25 (0.87, 1.45) | 0.375 | NS (excluded) | |
| Woodland (categorical, 1%) | | | | | |
| <10.87 | 1.83 (1.19, 2.45) | | | | |
| 10.87-16.70 | 2.56 (1.80, 3.31) | | | | |
| >16.70 | 3.06 (2.27, 3.85) | | | | |
| Woodland (continuous, 10%) | | 2.58 (1.51, 4.41) | <0.001 | 2.31 (1.28, 4.16) | 0.028 |
| Grassland (categorical, 1%) | | | | | |
| <14.13 | 1.75 (1.21, 2.36) | | | | |
| 14.13-27.38 | 2.65 (2.07, 3.23) | | | | |
| >27.38 | 3.91 (2.87, 4.95) | | | | |
| Grassland (continuous, 10%) | | 1.66 (1.38, 1.99) | <0.001 | 1.21 (1.15, 1.47) | 0.006 |
| Water body (categorical, 1%) | | | | | |
| <1.63 | 2.38 (1.34, 3.41) | | | | |
| 1.63-2.57 | 2.56 (1.60, 3.52) | | | | |
| >2.57 | 2.53 (1.99, 3.08) | | | | |
| Water body (continuous, 10%) | | 0.59 (0.08, 4.43) | 0.609 | NS (excluded) | |

| | | | | | |
|---|---|---|---|---|---|
| Urban land (categorical, 1%) | | | | | |
| <1.64 | 3.15 (2.49, 3.82) | | | | |
| 1.64-5.84 | 3.00 (2.05, 3.96) | | | | |
| >5.84 | 1.83 (1.57, 2.09) | | | | |
| Urban land (continuous, 10%) | | 0.65 (0.57, 0.74) | <0.001 | 0.83 (0.67, 0.96) | 0.036 |
| Rural residential areas (categorical, 1%) | | | | | |
| <5.06 | 2.95 (2.21, 3.69) | | | | |
| 5.06-7.75 | 2.41 (1.52, 3.29) | | | | |
| >7.75 | 1.82 (1.31, 2.33) | | | | |
| Rural residential areas (continuous, 10%) | | 0.36 (0.18, 0.70) | 0.003 | NS (excluded) | |
| Elevation (categorical, m) | | | | | |
| <70.53 | 0.83 (0.57, 1.09) | | | | |
| 70.53-115.98 | 2.80 (2.12, 3.49) | | | | |
| >115.98 | 3.56 (2.68, 4.44) | | | | |
| Elevation (continuous, 100m) | | 3.85 (2.54, 5.84) | <0.001 | NS (excluded) | |
| Bird distance (categorical, m) | | | | | |
| <61.72 | 1.83 (0.89, 2.77) | | | | |
| 61.72-78.22 | 2.87 (2.24, 3.50) | | | | |
| >78.22 | 2.44 (1.60, 3.27) | | | | |
| Bird distance (continuous, 10m) | | 1.06 (0.99, 1.13) | 0.091 | NS (excluded) | |
| Population density (categorical, 1/km2) | | | | | |
| <377.93 | 3.34 (2.51, 4.17) | | | | |
| 377.93-891.71 | 2.94 (2.22, 3.67) | | | | |
| >891.71 | 0.83 (0.57, 1.09) | | | | |
| Population density (continuous, 100/km2) | | 0.96 (0.94, 0.97) | <0.001 | NS (excluded) | |

| | | | | | |
|---|---|---|---|---|---|
| Cattle density (categorical, head/km2) | | | | | |
| <3.29 | 2.64 (1.63, 3.66) | | | | |
| 3.29-6.81 | 3.00 (2.32, 3.69) | | | | |
| >6.81 | 1.44 (1.01, 1.86) | | | | |
| Cattle density (continuous, 10 head/km$^2$) | | 0.95 (0.74, 1.42) | 0.129 | NS (excluded) | |
| Goat density (categorical, head/km2) | | | | | |
| <2.57 | 1.16 (0.73, 1.60) | | | | |
| 2.57-18.62 | 2.33 (1.73, 2.92) | | | | |
| >18.62 | 4.14 (3.14, 5.14) | | | | |
| Goat density (continuous, 10 head/km$^2$) | | 1.61 (1.40, 1.87) | <0.001 | 1.49 (1.12, 1.76) | 0.016 |
| Orchard (categorical, 1%) | | | | | |
| <6.01 | 1.60 (1.08, 2.12) | | | | |
| 6.01-14.87 | 2.96 (2.27, 3.65) | | | | |
| >14.87 | 2.64 (1.63, 3.66) | | | | |
| Orchard (continuous, 10%) | | 1.03 (0.81, 1.30) | 0.836 | NS (excluded) | |
| Tick SFTSV infection rate (categorical, 1%) | | | | | |
| <0.74 | 1.70 (0.55, 2.85) | | | | |
| 0.74-1.33 | 2.58 (0.40, 4.76) | | | | |
| >1.33 | 5.37 (4.13, 6.61) | | | | |
| Tick SFTSV infection rate (continuous, 1%) | | 2.74 (1.63, 4.62) | <0.001 | 1.65 (1.12, 2.43) | 0.011 |

Note: Variables were categorized by tri-sectional quantiles to display the average SFTS incidences at different levels. NS means variables were not significant in multivariate analysis. *P* values <0.05 were considered to indicate statistically significant differences.

**Supplementary Table S4. Age- and sex-stratified case numbers and fatality rates of SFTS patients in Yantai, 2013 – 2024.**

| Age group | Sex | Cases | Deaths | CFR (%) | *P* |
|---|---|---|---|---|---|
| Total | Male | 927 | 101 | 10.90 | 0.003 |
| Total | Female | 1037 | 73 | 7.04 | |
| ≥ 60 | Total | 1387 | 148 | 10.67 | - |
| ≥ 60 | Male | 681 | 88 | 12.92 | 0.009 |
| ≥ 60 | Female | 706 | 60 | 8.50 | |
| 40–59 | Total | 540 | 26 | 4.81 | - |
| 40–59 | Male | 222 | 13 | 5.86 | 0.459 |
| 40–59 | Female | 318 | 13 | 4.09 | |
| 0–39 | Total | 37 | 0 | 0 | - |
| 0–39 | Male | 24 | 0 | 0 | - |
| 0–39 | Female | 13 | 0 | 0 | |

Note: Data are presented as counts and case fatality rates (CFR, calculated as [death cases / total cases in the stratum] × 100%). The $\chi^2$ test was used to compare CFRs between males and females within each age group. *P* values less than 0.05 were considered statistically significant.

**Supplementary Figure S1. Median age of SFTS cases in Yantai City.**

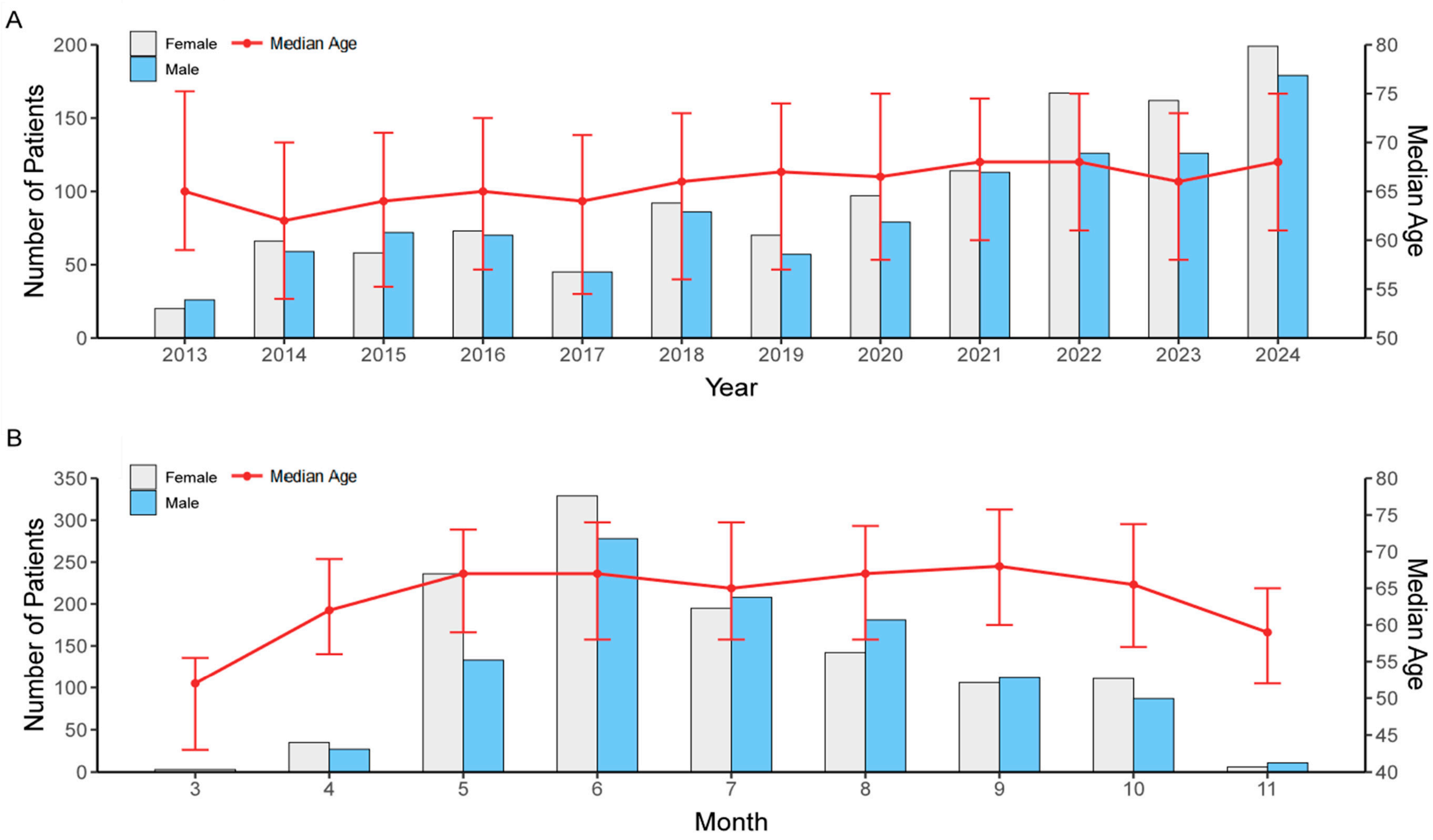


Note: (A) Median age of SFTS cases in different years. (B) Median age of SFTS cases in different months. The dots and the error bars denote median age and interquartile ranges. SFTS, severe fever with thrombocytopenia syndrome.

**Supplementary Figure S2. The delay days between disease onset and admission of SFTS cases in Yantai City.**

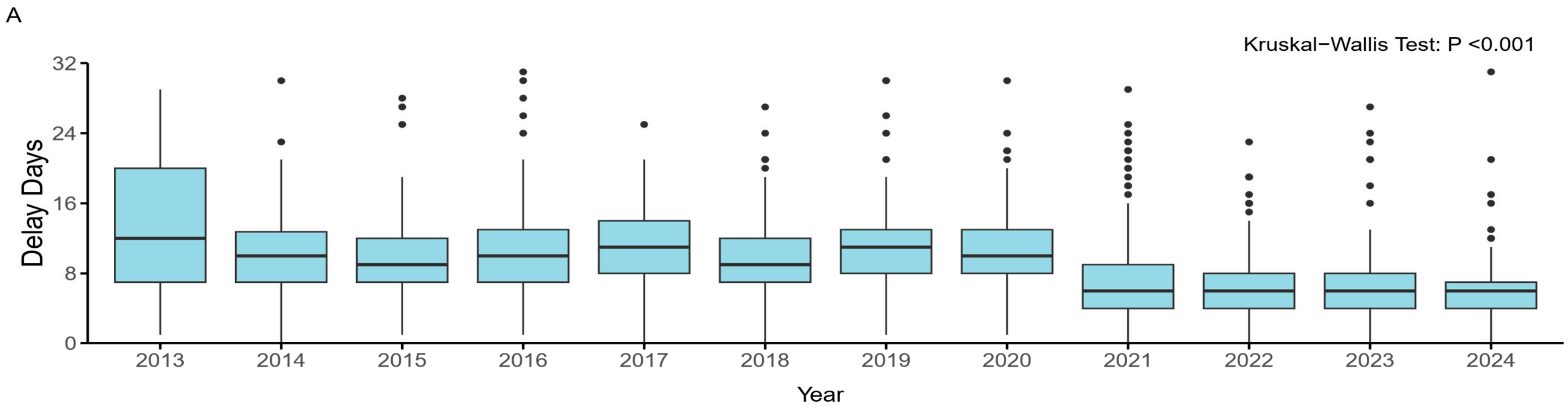


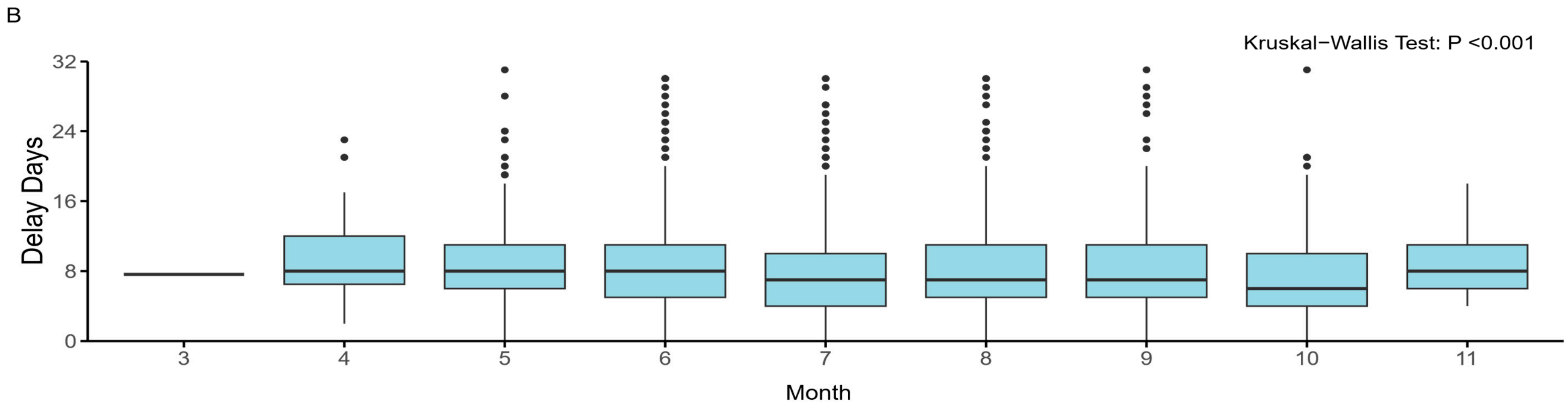


Note: (A) The comparison of delay days between disease onset and admission of SFTS cases in different years. (B) The comparison of delay days between disease onset and admission of SFTS cases in different months. SFTS, severe fever with thrombocytopenia syndrome. The comparison of delay days between different years or months used the Kruskal-Wallis test, *P* values <0.05 indicates statistically significant.

**Supplementary Figure S3. Monthly distribution of SFTS cases in every year.**

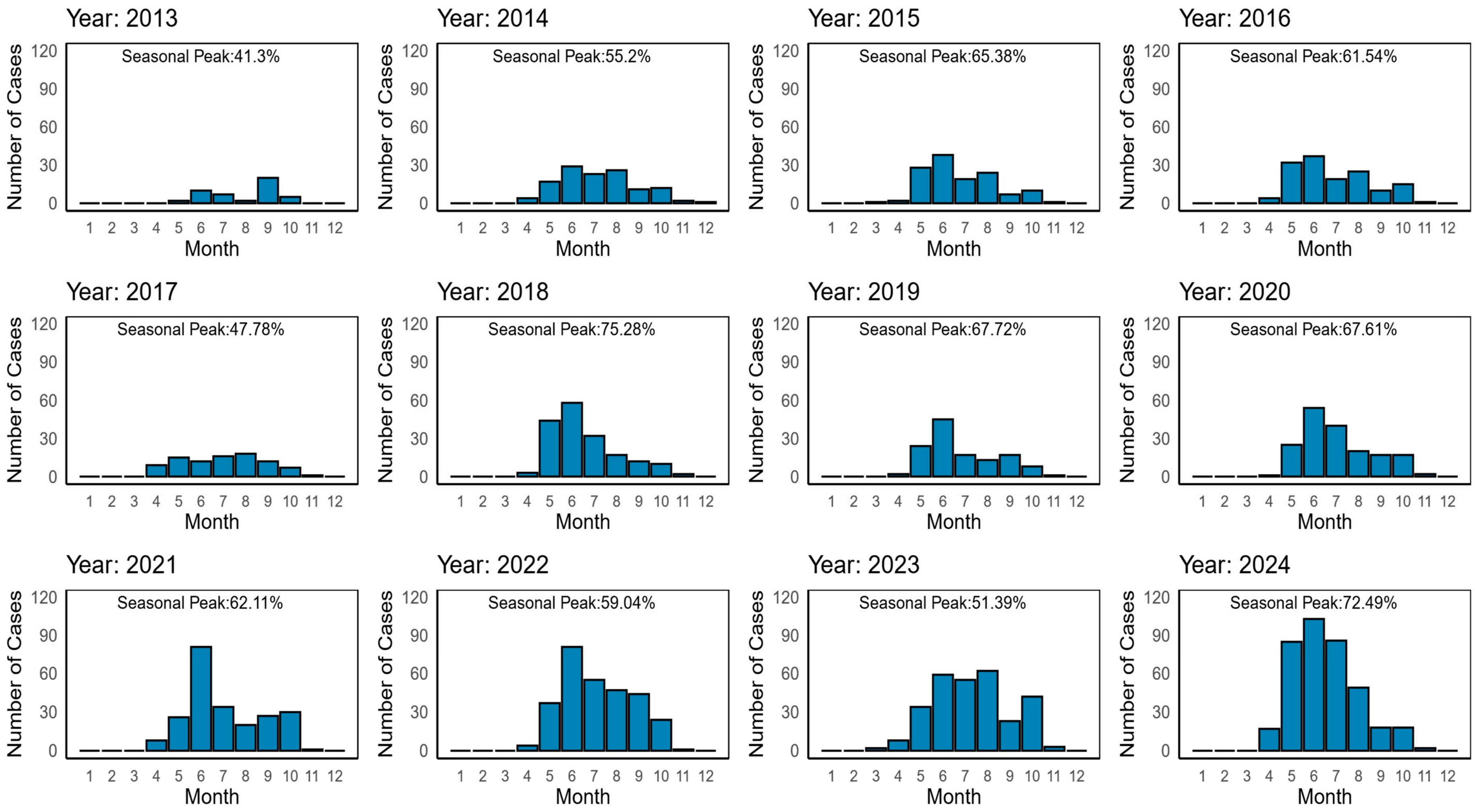


Note: SFTS, severe fever with thrombocytopenia syndrome. The percentage refers to the proportion of the number of cases from May to August accounting for the total number of cases throughout the year.

**Supplementary Figure S4. Average Annual Percent Change (APC) of SFTS incidence by bimonthly season in Yantai City, 2013－2024.**

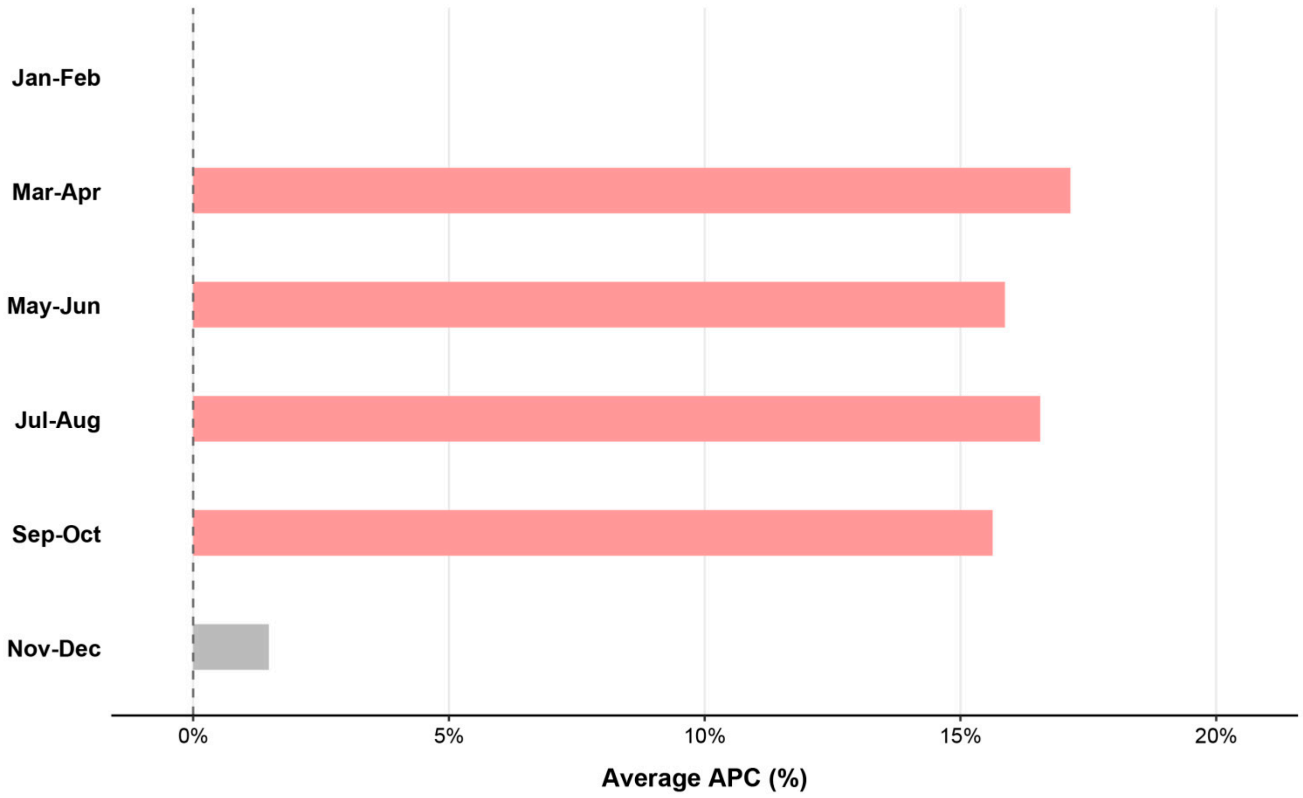


Note: Annual percentage change (APC) values were estimated using Joinpoint regression analysis. Red bars indicate periods with a statistically significant increasing trend (*P* < 0.05). Gray bars indicate periods with no statistically significant trend. SFTS, severe fever with thrombocytopenia syndrome.

**Supplementary Figure S5. Monthly trends in SFTS incidence by age group and sex in Yantai City, 2013－2024.**

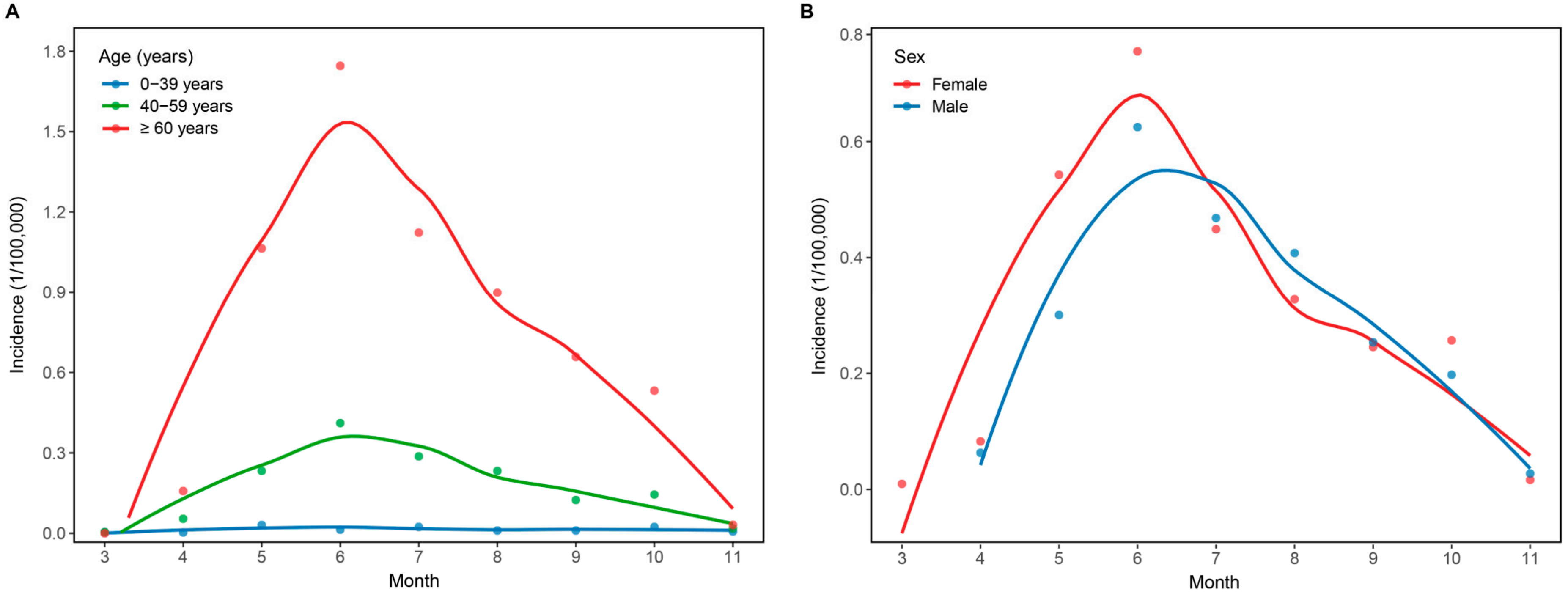


Note: SFTS, severe fever with thrombocytopenia syndrome. Loess-smoothed trends of monthly incidence rates are shown separately by age group (A) and sex (B). Age groups are 0-39, 40-59, and ≥ 60 years.

**Supplementary Figure S6. Correlation analysis between average SFTS incidence and environmental factors in Yantai City.**

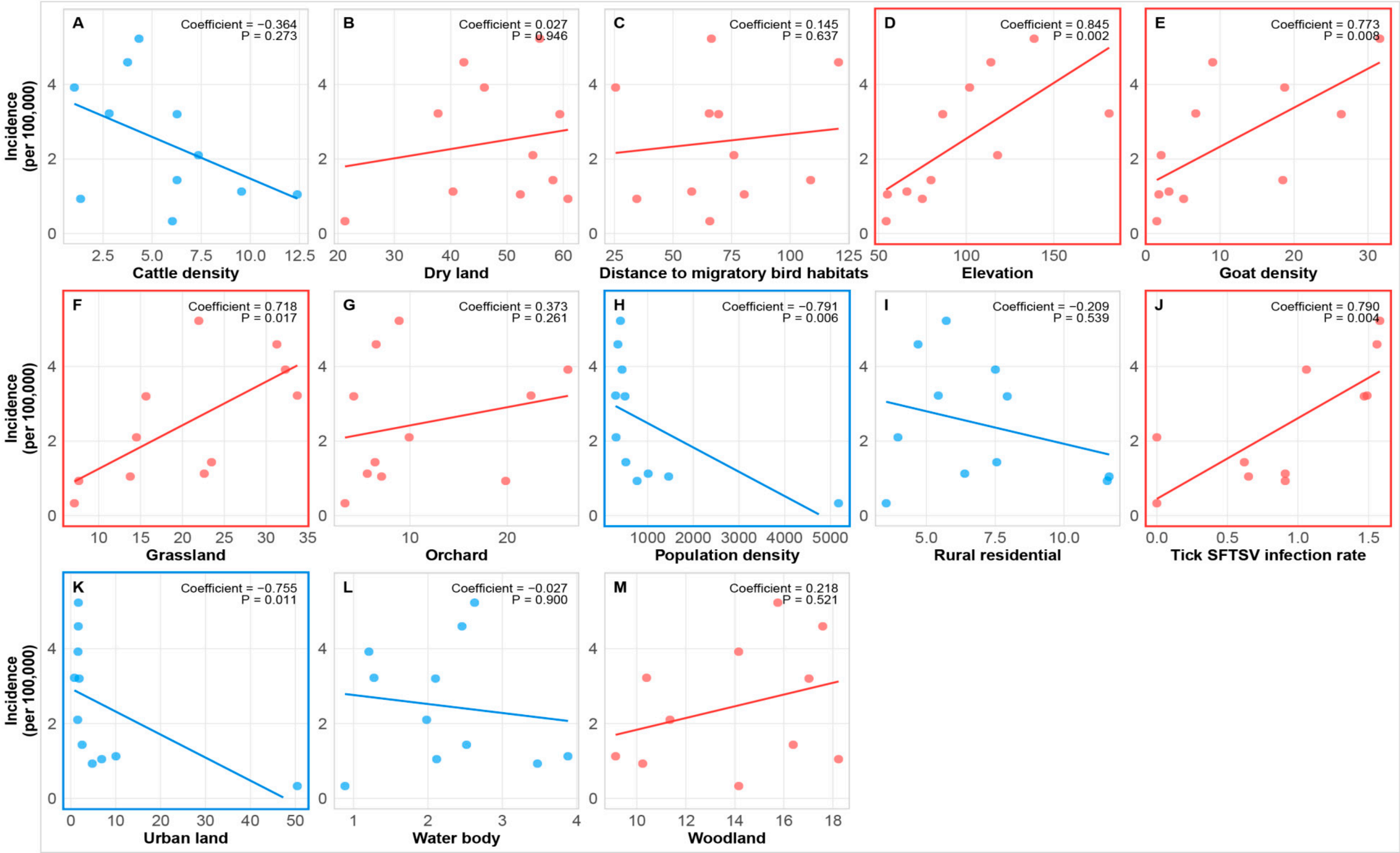


Note: SFTS, severe fever with thrombocytopenia syndrome. The red (positive correlation) or blue (negative correlation) lines represent linear regression fits. The Spearman rank correlation coefficient (rho) and corresponding *P* value are displayed in each subplot. *P* values <0.05 indicates statistically significant.

**Supplementary Figure S7. Temporal association between human SFTS incidence and SFTSV infection rate in H.longicornis ticks in Yantai City, 2019－2024.**

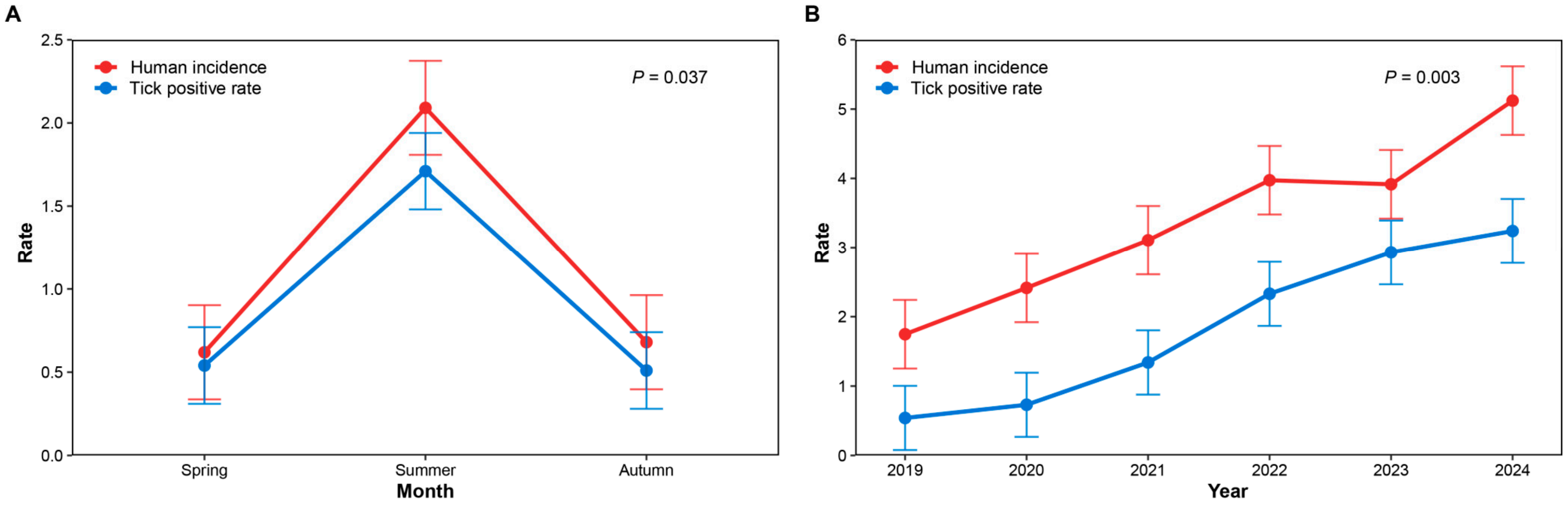


Note: (A) Seasonal patterns of human SFTS incidence and tick SFTSV infection rate. Seasonal data are aggregated as spring (March-May), summer (June-August), and autumn (September-November). (B) Annual trends of human SFTS incidence and tick SFTSV infection rate from 2019 to 2024. Points and error bars represent the rate and 95% confidence intervals, respectively. Spearman's rank correlation coefficients (ρ) and P-values for the annual and seasonal associations are presented, respectively. SFTS, severe fever with thrombocytopenia syndrome; SFTSV, SFTS virus.

**Supplementary Figure S8. Annual demographic characteristics of fatal SFTS cases in Yantai City, 2013 – 2024.**

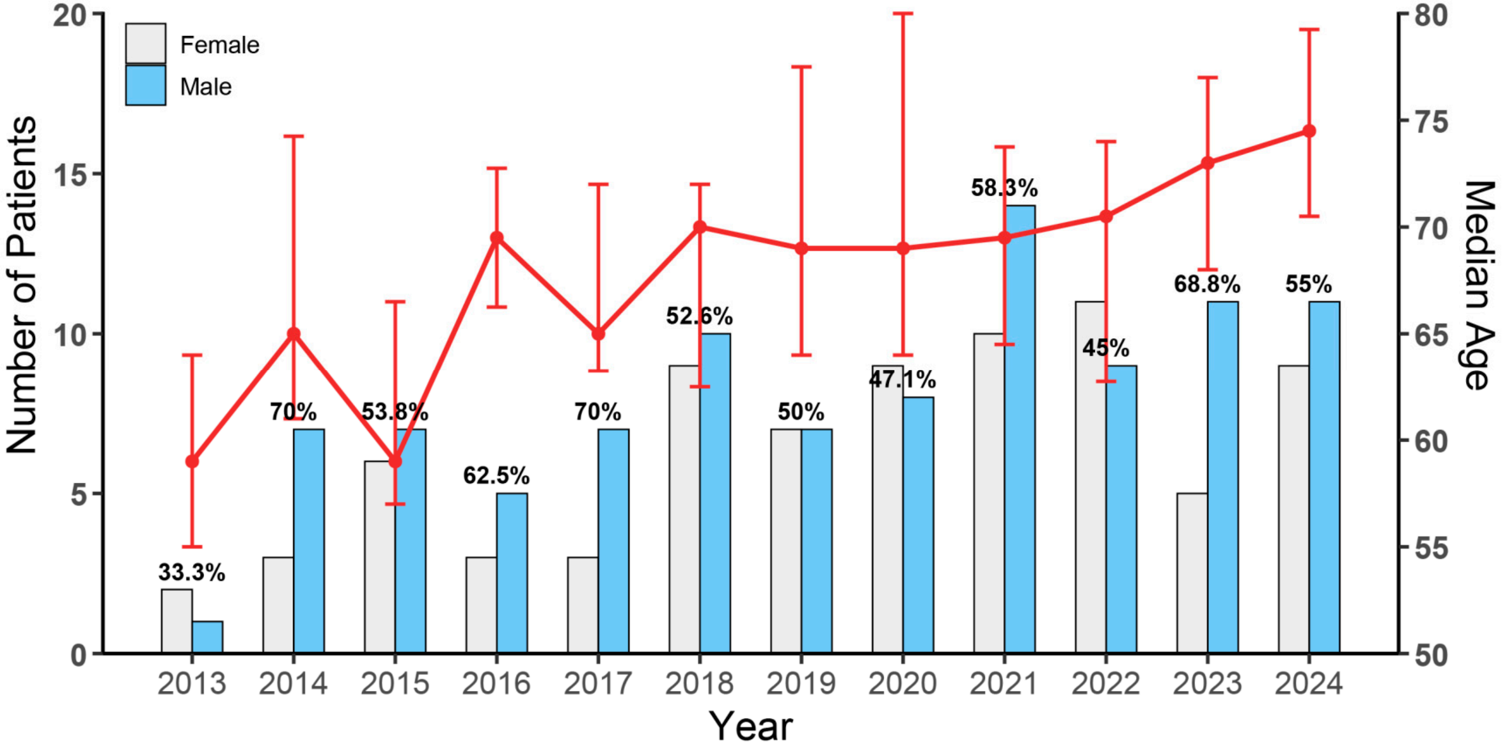


Note: SFTS, severe fever with thrombocytopenia syndrome.The bar chart shows the annual number of fatal SFTS cases stratified by sex (female in gray, male in light blue). Numbers above the bars represent the percentage of male deaths among total fatal cases for that year. The red line with points and error bars depicts the median age (with interquartile range, IQR) of fatal cases each year.

**Supplementary Figure S9. Annual case-fatality rates of SFTS by age group in Yantai City, 2013 – 2024.**

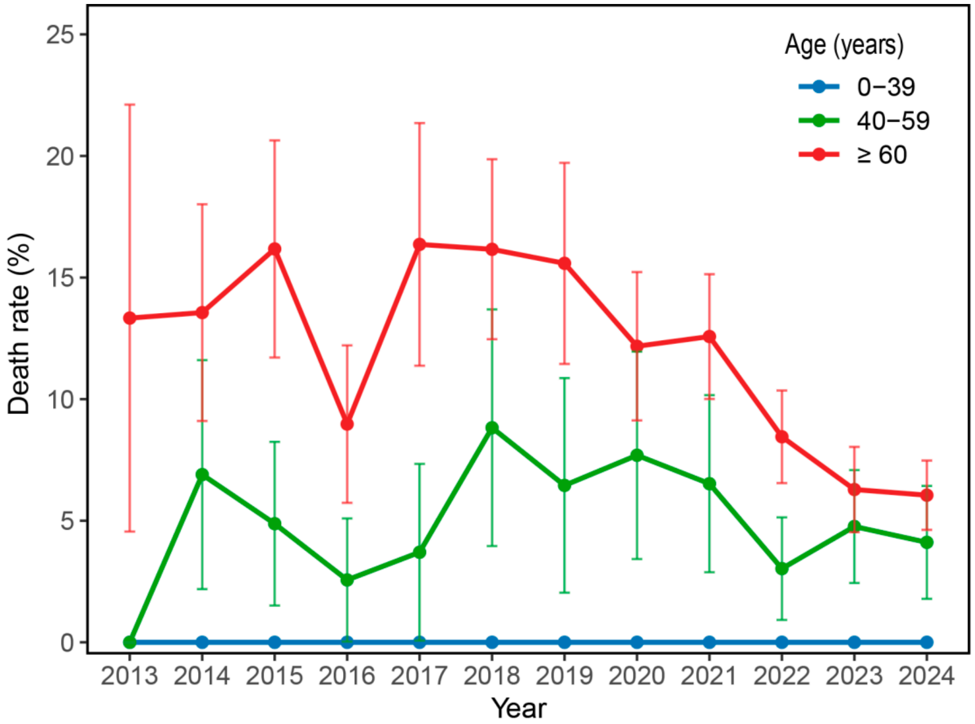


Note: SFTS, severe fever with thrombocytopenia syndrome. Case-fatality rates (%) are calculated as the proportion of fatal cases among all confirmed SFTS cases within each age group (0-39, 40-59, and ≥ 60 years) for each year. Data points represent annual case-fatality rates and bars indicate 95% confidence intervals.
